# Neurocognitive development of memory for landmarks

**DOI:** 10.3389/fpsyg.2015.00224

**Published:** 2015-03-06

**Authors:** Janneke van Ekert, Joost Wegman, Gabriele Janzen

**Affiliations:** ^1^Behavioural Science Institute, Radboud University Nijmegen, Nijmegen, Netherlands; ^2^Donders Institute for Brain, Cognition and Behaviour, Radboud University Nijmegen, Nijmegen, Netherlands

**Keywords:** cognitive development, spatial memory, navigation, landmarks, medial temporal lobe, anterior cingulate cortex

## Abstract

The capacity to detect landmarks in the environment and to associate each landmark with its spatial context is a fundamental operation for navigation, especially when the context is relevant for successful navigation. Recent evidence suggests robust age-related improvements in contextual memory. The current study investigated the effect of spatial context on landmark recognition memory in children and adolescents. Participants, ages 8–18, watched a video depicting a route through a virtual environment. The location at which landmarks occurred was manipulated to test the hypothesis that memory processes vary as a function of context. Functional magnetic resonance imaging data was acquired while participants performed an old-new recognition memory test of the landmarks. Old compared to new landmarks recruited a network of regions including the hippocampus and the inferior/middle frontal gyrus in all participants. Developmental differences were observed in the functional organization of the parahippocampal gyrus and the anterior cingulate cortex, such that memory representations strengthened linearly with age only when the associated spatial context was relevant for navigation. These results support the view that medial temporal lobe regions become increasingly specialized with development; these changes may be responsible for the development of successful navigation strategies.

## INTRODUCTION

Spatial navigation is a core characteristic of human behavior. It requires the ability to detect and memorize critical features in the environment, such as reference points or landmarks. Typically, when we travel a route through an unknown area, we remember *what* we encountered (i.e., a landmark) as well as phenomenological details such as *when* and *where* we encountered it. In a behavioral study, Janzen (2006) showed that the capacity to retain not only landmarks in memory but also the associated spatial context supports navigation. Recently, neuroscientists have established a direct link between age-related improvements in contextual memories and developmental changes in medial temporal lobe (MTL) function (Ghetti et al., 2010; DeMaster and Ghetti, 2013). However, the effect of spatial context on the neural representation of landmark recognition memory in children and adolescents remains poorly understood.

Studies on the neural correlates of navigation have delineated a role for the MTL in spatial memory and navigation. It has conclusively been shown that the hippocampus underpins the ability to encode and retrieve spatial locations within an environment (O’Keefe and Nadel, 1978; Burgess et al., 2002; Hassabis et al., 2009; Baumann et al., 2010). The parahippocampal gyrus (PHG) has been demonstrated to play a role in the processing of spatial scenes (Epstein and Kanwisher, 1998; Epstein, 2008) and in the encoding and retrieval of objects in large-scale environments (Aguirre et al., 1996; Maguire et al., 1998; Shelton and Gabrieli, 2002; Düzel et al., 2003; Rosenbaum et al., 2004). Neuroscientists interested in questions concerning the role of objects in navigation have highlighted the relevance of spatial context. Objects rarely occur in isolation; rather, they appear within a visual scene. The process of binding an object to the associated spatial context has likewise been ascribed to the MTL (Eichenbaum and Cohen, 2001; Diana et al., 2007; Oliva and Torralba, 2007; Aminoff et al., 2013). Specifically, Aminoff et al. (2013) emphasized the importance of the posterior part of the PHG in the formation of item-context associations.

Of particular relevance to the current study, neuroimaging studies in adults have revealed that the PHG is sensitive to the spatial context in which objects or landmarks are encountered (Janzen and van Turennout, 2004; Janzen et al., 2007, 2008; Janzen and Jansen, 2010; Schinazi and Epstein, 2010; Wegman and Janzen, 2011). In a series of studies, the location at which landmarks occurred was systematically manipulated to test the hypothesis that memory processes vary as a function of the associated context. Results indicated that landmarks encountered at a navigationally relevant location (i.e., an intersection referred to as decision point; DP) engaged the PHG during encoding and subsequent retrieval. No such a response was observed for landmarks encountered at an irrelevant location (i.e., a simple turn referred to as non-decision point; NDP).

The neural distinction between landmarks associated with a relevant spatial context and landmarks associated with an irrelevant spatial context is a major but not exclusive component of a mechanism underlying navigation. In order for a mechanism to be effective, it needs to additionally distinguish between associations that are helpful and associations that are misleading or ambiguous. Imagine a bus stop at a typical downtown intersection. The bus stop conveys spatial information that can be used to guide navigation. However, if a similar bus stop is located at another intersection along the route, the information provided is misleading or ambiguous. This is especially true when different behavior is required (e.g., turning left at the first bus stop and turning right at the second bus stop). In a recent study, the distinction was drawn between relevant and ambiguous landmarks (Janzen and Jansen, 2010). Ambiguous landmarks were operationally defined as identical landmarks situated at multiple relevant locations along the route. Employing a paradigm similar to that described in the previous paragraph, Janzen and Jansen (2010) demonstrated that ambiguous landmarks (i.e., objects located at two DP’s) did not recruit the PHG but instead activated a region in the prefrontal cortex (PFC) identified as the middle frontal gyrus. Taken together, strong evidence was found suggesting that the PHG is critical in the formation and retrieval of memory for landmarks when the associated spatial context supports successful navigation. Moreover, the PFC was shown to be essential in the representation of landmarks associated with an ambiguous spatial context.

In the past decades, researchers have sought to determine the developmental timeframe of landmark use. Behavioral studies have shown that the presence of landmarks facilitates navigation in 6-year-old children (Cornell et al., 1989; Jansen-Osmann and Fuchs, 2006). However, Jansen-Osmann and Fuchs (2006) pointed out that memory representations of landmarks differ for children and adults. Children form stable memories of landmarks, but unlike adults, they maintain only a weak association between landmarks and their spatial location in the environment.

While some research has been carried out on the development of memory for landmarks, there is little scientific understanding of the effect of spatial context on the neural representation of landmarks in children and adolescents. As memory for landmarks is dependent on fundamental operations in MTL and PFC regions, studies on functional changes in those regions may prove informative. In a review, Ofen (2012) put forward the idea that functional development of MTL and PFC regions varies as a function of the memory tested. Previous research has indicated that memory for objects is developed relatively early in life whereas development is protracted for tasks that require the ability to retain contextual information in memory (Cycowicz et al., 2001; Billingsley et al., 2002; Brainerd et al., 2004; Ofen et al., 2007; Ghetti and Angelini, 2008; Ghetti et al., 2010). In particular, Ghetti et al. (2010) demonstrated that in 8-year-olds, activation in MTL regions significantly predicted memory for objects regardless of whether contextual details were successfully recalled. In 10–11-year-olds the pattern of results was inconsistent, suggesting that children of this age group fell in a transitional stage. In 14-year-olds and adults, activation profiles in the hippocampus and PHG were predictive of context memory, but not memory for objects. Subsequent neuroimaging studies on the development of memory revealed that functional changes associated with developmental gains in contextual memory are not restricted to MTL regions but can also be observed in the PFC. For instance, Ofen et al. (2007) found that memory performance significantly improved with age when memories were accompanied by a vivid recollection of contextual details. By contrast, memory for objects showed little change with age. These behavioral results paralleled age-related changes in brain function: PFC activations associated with successful memory for contextual details grew steadily from age 8 to 24, whereas MTL activations associated with successful memory for objects remained constant across this age span.

Taken together, these results suggest that memory for objects is fully developed before the age of 8. In contrast, contextual memories follow a protracted course of development in both MTL and PFC regions. In the same vein, a prolonged developmental trajectory may be observed for the effect of spatial context on the neural representation of landmarks. The aim of the current study was to test this hypothesis.

**FIGURE 1:**
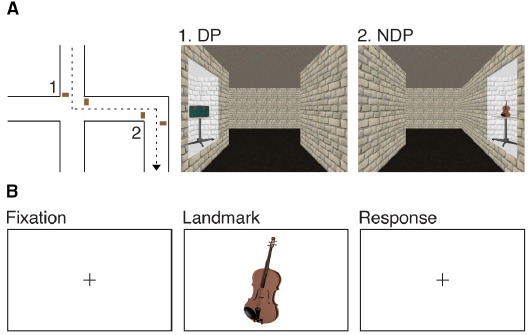
**Experimental design. (A)** Participants watched a film sequence through a large-scale virtual environment. They were instructed to remember the route and the objects along the route. Half of the objects occurred once at a DP or a NDP and half of the objects occurred twice at a DP or a NDP. **(B)** Participants performed an old-new recognition task of the objects while brain activity was measured using fMRI.

To accomplish this goal, children and adolescents, aged 8–18, watched a video depicting a route through a large-scale virtual environment (Figure 1A). They were instructed to remember both the route and the objects along the route. The location at which objects occurred was manipulated to test the hypothesis that memory processes vary as a function of the associated spatial context. Half of the objects occurred once at a DP or NDP and half of the objects occurred twice at a DP or NDP, the latter half providing a measure of ambiguity. Directly after the study phase participants performed an old-new recognition memory test on the objects (Figure 1A) while brain activity was measured using fMRI.

Memory for landmarks is hypothesized to be well developed before the age of 8. This is thought to be manifested by sustained activation in MTL regions for old versus new objects in all children and adolescents. Second, a prolonged maturational trajectory is expected for effects of spatial context on the neural representation of landmarks. We hypothesize an increase with age in the posterior part of the PHG for landmarks associated with a relevant spatial context compared to landmarks associated with an irrelevant or ambiguous spatial context. Similarly, we hypothesize an increase with age in PFC activity for landmarks associated with an ambiguous spatial context.

## MATERIALS AND METHODS

### PARTICIPANTS

Thirty-nine volunteers, ages 8–18 (13.89 ± 3.18), were recruited from the local community. Together with their caregivers they provided informed consent according to the Declaration of Helsinki prior to participating in the study. Data from four participants (three 18-year-old females, one 10-year-old male) were excluded for the reason that technical difficulties occurred when collecting data. In addition, data from two participants (two 18-year-old females) were discarded from analyses due to anomalous findings. Data from one participant (one 13-year-old female) were excluded as a result of anxiety during fMRI data acquisition. Consequently, 32 participants (37.5% male) contributed to the final sample (children, ages 8–11, *n* = 10; adolescents, ages 12–15, *n* = 12; young adults, ages 16–18, *n* = 10). The gender of the participants was not related to age (*r_pb_* = 0.02, *P* = 0.911), rendering it unlikely that gender was a confounding factor in the current study.

Participants had normal or corrected-to-normal vision and reported no history of neurological impairment. Caregivers filled out the child behavior checklist (CBCL) in order to screen for psychiatric conditions (Achenbach et al., 1991). With the exception of one participant, all scored below clinical levels of the CBCL, and had scores within one SD of the mean of a normative standardized sample. One 17-year-old male participant scored within two SD of the mean. Participants completed the Raven-Standard Progressive Matrices (R-SPM) test (Raven, 1941). The R-SPM assesses cognitive functioning by means of a visuospatial task that requires participants to identify the missing item that completes the stimulus pattern. The outcome provides a reliable estimate of intelligence. A negative trend was observed in the relation between estimated intelligence quotient (IQ) scores and age (*r* = –0.33, *P* = 0.063), indicating that in this sample IQ scores decreased with age. Children received a gift certificate for their participation. The study was approved by the CMO committee on Research Involving Human Participants (Region Arnhem-Nijmegen).

### MATERIALS AND PROCEDURE

The study took place on two separate days. During the first day of assessment, participants completed the R-SPM and caregivers filled out the CBCL. Thereafter, participants were accustomed to imaging procedures in a MRI mock scanner. The set-up of the mock scanner consisted of a simulation scanner with visual and auditory presentation systems and equipment for monitoring participants’ responses. Participants were exposed to noises that are typical of MRI data acquisition. In addition, they practiced to lie still in confined space. Subsequently, participants were trained on a simple computer task for the purpose of familiarization with fMRI research. Each trial of the computer task consisted of a fixation cross, followed by a picture of an animal on a white background. By button presses, participants indicated as quickly and as accurately as possible whether there was one or more than one animal in the picture. Although these procedures aim at training young children, all participants (including 18-year olds) were familiarized with the MRI environment to maintain methodological consistency.

The second day of assessment was divided into two parts: an encoding phase and a retrieval phase during which functional images of the brain were acquired (Figure 1). Participants took part in all conditions of the encoding and retrieval phase, indicating that a repeated measures design was employed. During the encoding phase, participants watched a film sequence of a tour through a virtual environment. They received the following standardized instruction: “You will be guided through a virtual museum that exhibits all kinds of objects. These objects are placed on tables along the wall. Assume you are asked to guide your fellow students through the museum later today. Therefore, while you are watching the film, try to memorize the route and the objects along the route.”

Blender 2.49b^1^ was used to create the virtual environment from which the film sequence was recorded. The environment had a maze-like layout, consisting of straight corridors alternated with intersections (DP) and simple turns (NDP). Corridors were 3.3 m wide, 3.3 m high and 23.1 m long. We selected 120 three dimensional models of common objects. Those objects appeared on tables along the wall, either directly before or after a turn was made. In the environment, half of the objects occurred once at a DP or a NDP and half of the objects occurred twice at a DP or a NDP. Thus, four conceptually distinct conditions were introduced, namely: a condition in which objects occurred once at a DP (1DP, 30 objects), a condition in which objects occurred once at a NDP (1NDP, 30 objects), a condition in which objects occurred twice at a DP (2DP, 30 objects) and a condition in which objects occurred twice at a NDP (2NDP, 30 objects). Right and left turns were counterbalanced over conditions. If an object occurred twice in the environment, either a similar (right–right or left–left) or a different (right–left or left–right) turn was made. Similar turns appeared less often (33%) as compared to different turns (67%) to probe the sense of ambiguity. Each object remained visible for 3.3 s on average. The viewpoint moved through the environment at a simulated eye-level of 1.65 m, at a constant speed of 3.58 km/h. The film sequence was split into half, each part lasting 12.13 min. The presentation order of the two film sequences was counterbalanced over participants.

Following the encoding phase, participants performed a recognition memory task inside the scanner. We selected an additional 90 three dimensional models of common objects to serve as lures. Consideration was given to the unequal number of experimental items and lures. In line with previous studies (Ghetti and Angelini, 2008; Ghetti et al., 2010; DeMaster and Ghetti, 2013), significantly less lures were included in the current study to minimize the burden on participants. Two dimensional screen shots were taken of both experimental and distracter items to meet the requirements of the old-new recognition memory test. Each trial consisted of a fixation cross, followed by an object shown on a white background for 500 ms. Thus, during scanning, no context-related information was presented. All stimuli were presented rapidly, in a randomly intermixed order to prevent participants from anticipating and changing strategies for the different event types. By button presses, participants indicated as quickly and as accurately as possible whether the object had occurred in the former film sequences. The average inter-stimulus interval was 5000 ms, jittered between 4000 and 6000 ms in steps of 250 ms, counterbalanced over conditions.

### IMAGE ACQUISITION

Functional images of the whole brain were acquired on a 3T Siemens Trio scanner (Siemens, Erlangen, Germany). We used a gradient-echo planar scanning sequence to collect 31 axial slices (voxel size 3 × 3 × 3 mm^3^, TR = 2000 ms, TE = 30 ms, field of view = 208 mm, flip angle = 75°). Following acquisition of the functional images, we acquired a high resolution T1 weighted anatomical scan (MP-RAGE; 192 sagittal slices, TR = 2300 ms; TE = 3.03 ms; 8° flip angle; slice thickness = 1 mm; FOV = 256 mm; GRAPPA parallel imaging with an acceleration factor of 2). Finally, we collected diffusion-weighted data which will be published elsewhere.

### IMAGE PROCESSING AND DATA ANALYSIS

Behavioral data was analyzed using repeated measures MANCOVA with recognition memory performance (probability of a hit) and response time (in milliseconds) as dependent variables, spatial context (1DP, 1NDP, 2DP, 2NDP) as an independent variable and age (in years) as a covariate.

fMRI data were preprocessed and analyzed with SPM8^2^. The first five volumes of each participant’s EPI data were discarded from analyses to allow for T1 equilibration. The functional images were slice time corrected, and the subject mean was coregistered with the corresponding T1 weighted structural scan using normalized mutual information optimization. The structural image was segmented into gray matter, white matter and cerebrospinal fluid, functional images were spatially normalized and transformed into common space, as defined by the Montreal Neurological Institute (MNI) T1 template, a procedure common to developmental neuroimaging research (Crone et al., 2010). Finally, the images were spatially filtered by convolving them with an isotropic 3D Gaussian kernel (6 mm full width at half maximum).

Statistical analyses were performed within the framework of the general linear model (GLM). Regressor functions were constructed for five different event types (1DP, 1NDP, 2DP, 2NDP and lures). The following contrasts of interest were defined: (i) contrast 1DP + 1NDP + 2DP + 2NDP > lures; (ii) contrast 1DP > 1NDP; (iii) contrast 2DP > 2NDP. Subsequently, individual subject’s effects were estimated. As detailed below, the contrast images were input into second-level group analyses using one-sample *t*-tests to examine the neural representation of memory for landmarks and simple regression analyses to examine the effect of age (in years) on the neural representation of memory for the spatial context with which landmarks are associated. The results were initially thresholded at voxel level *P* = 0.001 (uncorrected) and the cluster-size statistics were used as the test statistic. Only clusters at *P* 0.05 (family-wise error corrected) were considered significant. Small volume correction was applied to the bilateral posterior PHG and the bilateral hippocampus (Lancaster et al., 2000). The results of those analyses were initially thresholded at voxel level *P* = 0.001 (uncorrected), and the correction was applied on the cluster-level for multiple comparisons (*P_FWE—SVC_* < 0.05). All local maxima are reported in MNI coordinates.

## RESULTS

### RECOGNITION MEMORY PERFORMANCE

Recognition memory performance was above chance (probability hit minus probability false alarm: Mean: 69.05%, *t*(31) = 23.69, *P* < 0.001, where 0% indicates participants performed at chance). Age was not predictive of false alarm rates (*b =* 0.53, *P* = 0.302), nor did it explain a significant proportion of the variance in false alarm rates [*F*(1,30) = 1.10, *P* = 302]. However, a marginally significant relationship was observed between the probability of a hit and age [*F*(1,30) = 3.43, *P* = 0.074], indicating that performance decreased proportionally with age. Moreover, the probability of a hit was not affected by the spatial context with which landmarks were associated [*F*(3,90) = 0.09, *P* = 0.963]. The absence of the effect of spatial context on memory performance was observed across the entire age span [*F*(3,90) = 0.98, *P* = 0.404].

A similar pattern of results was observed for the time needed to respond to a stimulus item. A significant relationship was observed between the probability of a hit and age [*F*(1,30) = 8.76, *P* = 0.006], indicating that response times decreased proportionally with age. Moreover, the spatial context with which landmarks were associated did not affect response times [*F*(3,90) = 0.04, *P* = 0.990]. The absence of the effect of spatial context on the time needed to respond to an object was observed across the entire age range [*F*(3,90) = 0.18, *P* = 0.912].

### DEVELOPMENT OF MEMORY FOR LANDMARKS

**FIGURE 2:**
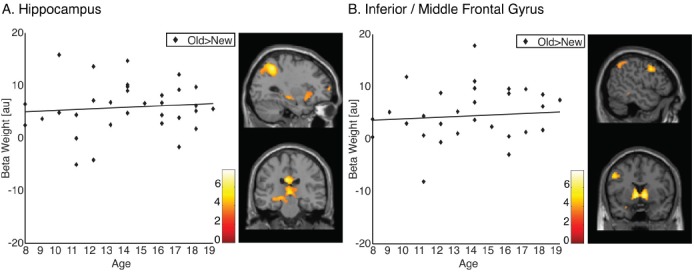
**Memory representations of landmarks. (A)** Old compared to new items (1DP + 1NDP + 2DP + 2NDP > lures) recruit the hippocampus in all participants. **(B)** Old compared to new items (1DP + 1NDP + 2DP + 2NDP > lures) recruit the inferior/middle frontal gyrus in all participants.

To examine the effect of age on the neural representation of memory for landmarks, we first identified activations that were greater for old items encountered in a virtual environment than for new items (contrast 1DP + 1NDP + 2DP + 2NDP > lures). Individual subject’s effects were input into group analyses using a one-sample *t*-test. Across all 32 participants, activations were found in the left hippocampus (x = –30 y = –22 z = –10, *k* = 11, *P_SVC_* = 0.027) as well as in the left inferior/middle frontal gyrus (x = –50 y = 8 z = 38, *k* = 272, *P* = 0.041; Figure 2A). In addition, large bilateral posterior clusters were observed spanning the angular gyrus, precuneus, posterior cingulate and the thalamus (Table 1).

**Table 1:** **Clusters of activation for the contrast 1DP + 1NDP + 2DP + 2NDP > lures**.

**Region**	**x**	**y**	**z**	**k**	**T score at peak**
Angular gyrus/precuneus	—36	—54	46	10652^***^	7.87
Posterior cingulate	—6	—30	26		7.62
Thalamus	—6	—6	4		7.21
Inferior/middle frontal gyrus	—50	8	38	272^*^	5.07
Medial temporal lobe ROI					
Left hippocampus	—30	—22	—10	11^*^	4.15

The x, y, z coordinates of local maxima are displayed in MNI standard space coordinates. Whole brain threshold P < 0.001, uncorrected. k = cluster size. *P < 0.05 at cluster level, **P < 0.01 at cluster level, ***P < 0.001 at cluster level.

To examine developmental changes in brain function, regionally averaged beta weights were extracted from functionally defined clusters of activation. Given the role of the PFC and the MTL in the formation of memory, functional ROI’s were restricted to the inferior/middle frontal gyrus and the hippocampus. Beta weights from these regions were subjected to correlation analysis. The data revealed that there were no correlations between age and activation in the hippocampus (*r* = 0.094, *P* = 0.305, one tailed) or between age and activation in the inferior/middle frontal gyrus (*r* = 0.085, *P* = 0.322, one tailed; Figure 2B). These results suggest that PFC and MTL regions which are frequently associated with memory do not undergo functional changes within the examined age range.

### NEURAL RESPONSES TO THE NAVIGATIONAL RELEVANCE OF SPATIAL CONTEXT

**FIGURE 3:**
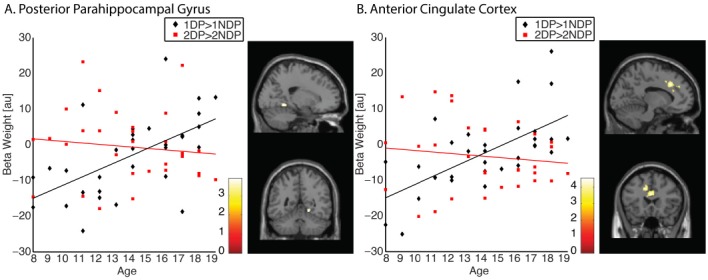
**Memory representations of landmarks are modulated by the navigational relevance of the associated spatial context. (A)** The representation of 1DP > 1NDP increased significantly with age in the right posterior PHG (x = 20 y = –50 z = –6). Contrary, no increase was observed in this region for the 2DP > 2NDP contrast. **(B)** The representation of 1DP > 1NDP increased significantly with age in the ACC (x = –12 y = 22 z = 36). Contrary, no increase was observed in this region for the 2DP > 2NDP contrast.

A similar approach was used to address the question whether age-related functional changes may be restricted to memory representations of landmarks associated with a spatial context relevant for navigation. To that end, we computed two contrasts. First, we identified clusters that demonstrated greater activation for landmarks associated with a relevant spatial context compared to landmarks associated with an irrelevant spatial context (contrast 1DP > 1NDP). Single subject’s effects were estimated and input into group analysis using the one-sample *t*-test. Across all participants, no significant clusters of activation were found. A comprehensive explanation that accounts for the current finding would be that the neural response to navigational relevance is reversed for children compared to adolescents. To test this assumption, individual subject’s effects were entered into simple regression analysis with age (in years) as predictor variable. Indeed, the results indicated that the selective response to landmarks associated with a relevant spatial context increased significantly with age in the right posterior PHG (x = 20 y = –50 z = –6, *k* = 39, *P_SVC_* = 0.042; Figure 3A). An identical pattern of results was observed in the anterior cingulate cortex (ACC), a region known to be involved in cognitive control (x = –12 y = 22 z = 36, *k* = 599, *P* = 0.002; Figure 3B). Second, we identified activations that were greater for landmarks encountered twice at a location relevant for navigation compared to landmarks encountered twice at a location irrelevant for navigation (contrast 2DP > 2NDP). Individual subject’s effects were input into group analyses using a one-sample *t*-test. Across all 32 participants, no significant clusters of activations were found. Subsequently, contrast images were entered in simple regression analysis with age (in years) as predictor variable. Results revealed no age-related increases in activation in the middle frontal gyrus, nor in any other regions of the brain. Taken together, these results imply that activation profiles in the PHG and ACC become more selective during development, such that these regions are engaged specifically for landmarks associated with a context relevant for navigation. There were no signs of evidence showing that the PFC is essential in the representation of landmarks associated with an ambiguous spatial context.

## DISCUSSION

The current study examined whether the neural representation of landmarks in the MTL and PFC changes during the childhood years. Children and adolescents, ages 8–18, watched a route through a large-scale virtual environment. The location of landmarks in the environment was systematically manipulated to test the hypothesis that the neural representation varies as a function of the associated spatial context. Subsequently, participants performed an old-new recognition memory test of the landmarks. Results indicated that old compared to new landmarks recruited a network of regions associated with successful memory formation in all participants, including the hippocampus and the inferior/middle frontal gyrus. This finding suggests that the neural representation of memory for landmarks develops early in age. Developmental differences were observed when a distinction was drawn between landmarks associated with a relevant spatial context and landmarks associated with an irrelevant spatial context. Memory representations in the PHG and ACC increased linearly with age only when the associated spatial context was relevant for navigation. No linear increases in PFC regions were observed for memory of landmarks associated with an ambiguous spatial context. These results imply that MTL but not PFC regions become increasingly sensitive to the navigational relevance of the spatial context in which landmarks are encountered. These changes may be responsible for the development of successful navigation strategies.

Memory can be thought of as the product of multiple components. For example, object memory requires the ability to recall a previously learned object as well as specific details about the context in which that object was learned. Testing memory components rather than testing memory as a unitary entity has gained increasing interest over the past decades. A developmental neuroimaging study by Ofen et al. (2007) examined developmental differences in memory through the use of a paradigm that required participants to reflect on their memory by making familiar/remembered judgments. Items being judged as familiar were thought to reflect object memory whereas items being judged as remembered were thought to reflect memories of objects that are accompanied by recollection of contextual details. They demonstrated no age-related changes in either memory or brain activation for memory that occurs without recollection of contextual details, suggesting that simple object memory is fully developed by the age of 8. This finding accords with our observation that old compared to new landmarks recruit a network of regions frequently associated with the formation of memory, including the hippocampus and the inferior/middle frontal gyrus. Pivotally, these activations remain stable throughout middle childhood and adolescence.

While simple object memory does not show improvements after the age of 8, the ability to retrieve memories that are accompanied by a vivid recollection of contextual details is marked by robust age-related changes until late adolescence (Chai et al., 2010; Raj and Bell, 2010; Ghetti and Bunge, 2012). Neuroscientists have argued that improvements in item-context associations are the result of age-related changes in MTL function. For instance, Ghetti et al. (2010) demonstrated that in 8-year-olds, activation in these regions significantly predicts memory for objects regardless of whether contextual information is successfully retrieved. In 14-year-olds and adults however, activation profiles in the hippocampus and PHG predict successful retrieval of the context, yet they do not predict memory for the object itself, suggesting that the ability to remember contextual details continues to develop after the age of 8. Here we provide evidence for developmental changes that are even more specific than was suggested by Ghetti et al. (2010). In the current study, developmental differences in the MTL occurred only when responding to landmarks associated with a spatial context relevant for navigation. Thus, the neural response to memory for landmarks develops early in age, yet MTL regions are subject to age-related changes in drawing a fine distinction between task relevant and task irrelevant spatial contexts.

One plausible account for developmental differences in MTL function comes from longitudinal work undertaken by Gogtay et al. (2006). They identified the nature as well as the developmental timeframe of hippocampal maturation. Whereas the overall size of the hippocampal formation was found to be relatively stable between ages 4 and 25 years, the posterior part increased in volume and the anterior part decreased. Although the driving force behind volumetric differences is not clear, some researchers maintain that the process of volume gain and loss reflects synaptic production, pruning and myelination (Utsunomiya et al., 1999). These processes may result in more reliable and specialized activation profiles and increased context memory performance (DeMaster and Ghetti, 2013). However, this assumption still needs to be tested.

A recent neuroimaging study in adults demonstrated the involvement of the PFC in spatial navigation (Janzen and Jansen, 2010). Employing a paradigm similar to the current study, they showed that landmarks which were situated at multiple relevant locations along a route activate a region in PFC identified as the right middle frontal gyrus. Surprisingly, no age-related increases in activation were observed in the PFC. Although the PFC is not essential for the formation of new memories, the ability to retain contextual information in memory has been ascribed to this region. For instance, initial studies in patients showed that prefrontal lesions impair declarative memory for contextual details of an experience (Schacter et al., 1984; Janowsky et al., 1989). From a developmental perspective, not much is known about the involvement of PFC regions in landmark processing. Yet, convergent evidence suggests that the PFC follows a prolonged maturational trajectory. Anatomical findings indicate that the structural architecture of the PFC changes with age until the early 20s (Paus et al., 1999; Giedd, 2004, 2008; Gogtay et al., 2004; Tsujimoto, 2008). This change is characterized by linear increases in white matter, and inverted U-shape changes in gray matter (Paus et al., 1999; Giedd, 2004, 2008; Gogtay et al., 2004; Tsujimoto, 2008). As a result of the immature state of PFC regions, it may be that the adolescent brain is unable to respond to landmarks associated with an ambiguous spatial context. Differences in the neural representation of ambiguous landmarks could possibly be observed in samples including older participants. Alternatively, it might be that due to the correlational design of this study the power to demonstrate an effect in older participants was not large enough. Adolescence is a period of rapid change which may result in temporary instability in brain networks. This generally results in large inter-individual differences which renders it unlikely to detect an effect.

Previous research employing a paradigm similar to the current study has conclusively demonstrated the involvement of the MTL in the representation of landmarks (Janzen and van Turennout, 2004; Janzen et al., 2007, 2008; Janzen and Jansen, 2010; Schinazi and Epstein, 2010; Wegman and Janzen, 2011). The results of the current study additionally point to a role for the ACC in landmark processing in children and adolescents. Successful memory for contextual details is generally associated with the capacity to monitor accuracy of retrieved information and the ability to detect performance errors. These operations are fundamental to cognitive control and are known to be supported by a well-delineated neural network of which ACC is a key component (Carter et al., 1998; Braver et al., 2001; Luna et al., 2010). Research establishing the link between cognitive control and context memory performance primarily stems from aging studies. In a systematic study by Spencer and Raz (1994) participants were administered a memory test in which they were asked to recall item and context information of previously learned facts. Results demonstrated that age had a greater effect on memory for context information compared to memory for item information. Importantly, poor performance on measures of cognitive control (e.g., Wisconsin Card Sorting Task; Grant and Berg, 1948; Heaton, 1981) were predictive of poor memory for contextual details, providing evidence for the premise that impairments in memorizing contextual information may depend on cognitive control functioning (Raj and Bell, 2010). Unfortunately, few studies have examined the relationship between cognitive control and context memory in children. Yet, it has been shown that cognitive control processes follow a protracted course in development (Raj and Bell, 2010). Likewise, the recruitment of the ACC significantly increases with age. In the current study, memory representations in the ACC increased with development. Crucially, the increase was restricted to memories for landmarks of which the associated spatial context was relevant for navigation. It may be that young children did not show any signs of neural sensitivity to the spatial context in which landmarks occur, due to the fact that these tasks require cognitive control processes. However, previous research in adults has shown that the associated spatial context is processed in an automatic rather than controlled manner (Janzen and van Turennout, 2004; Janzen et al., 2007, 2008; Janzen and Jansen, 2010; Schinazi and Epstein, 2010; Kessels et al., 2011; Wegman and Janzen, 2011). Therefore more research is needed to examine the role of the ACC in landmark recognition memory in children and adolescents.

To conclude, our findings have important implications for theory on the development of memory for landmarks. Previous research convincingly demonstrated age-related improvements in contextual memories. These improvements parallel changes in the functional organization of the MTL. Here, we provide evidence that age-related changes are much more specific than was previously thought. Both children and adolescents recruited a network of regions involved in the formation of memory for landmarks, suggesting that simple landmark memory develops early in life. Developmental differences occurred when a fine distinction was drawn between landmarks associated with a spatial context relevant for navigation and landmarks associated with a spatial context irrelevant for navigation. Between ages 8 and 18 years, activations in the posterior part of the PHG and the ACC increase linearly when the associated spatial context supports navigation. As such, we reveal a developmental timeframe for the establishment of a neural network that enables successful navigation.

## Conflict Of Interest Statement

The authors declare that the research was conducted in the absence of any commercial or financial relationships that could be construed as a potential conflict of interest.

## Footnotes

1 http://www.blender.org/>http://www.blender.org/">http://www.blender.org/

2 http://www.fil.ion.ucl.ac.uk/spm
